# Supplementary material for: Analysis of Ser/Thr Kinase HASPIN-Interacting Proteins in the Spermatids
Source: Int J Mol Sci. 2022 Aug 13;23(16):9060. doi: 10.3390/ijms23169060 (PMC9409403; doi:10.3390/ijms23169060)
Supplement: Supplementary file 1 [file ijms-23-09060-s001.zip › ijms-1835592-supplementary.pdf]

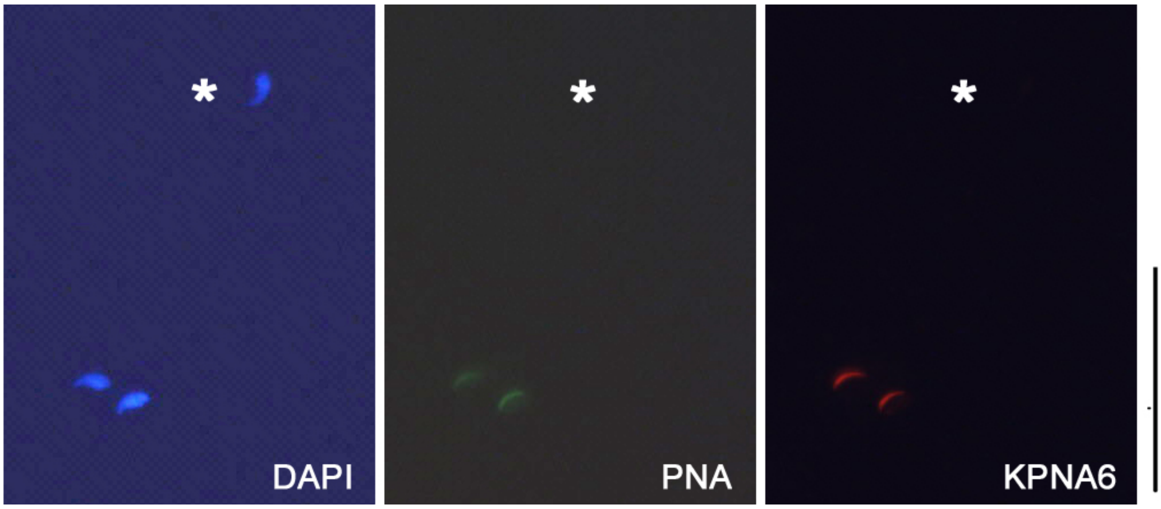

Figure S1 Sperm were stained with DAPI, FITC-PNA, the anti-KPNA6 antibodies. KPNA6 is not observed in sperm that have completed the acrosome reaction (star).Bar=50mM.
